# Supplementary material for: trRosettaRNA: automated prediction of RNA 3D structure with transformer network
Source: Nat Commun. 2023 Nov 9;14:7266. doi: 10.1038/s41467-023-42528-4 (PMC10636060; doi:10.1038/s41467-023-42528-4)
Supplement: Supplementary file 1 — Supplementary Information [file 41467_2023_42528_MOESM1_ESM.pdf]

Supplementary Materials for

trRosettaRNA: automated prediction of RNA 3D structure with transformer network

Wenkai Wang, Chenjie Feng, Renmin Han, Ziyi Wang, Lisha Ye, Zongyang Du, Hong Wei, Fa Zhang, Zhenling Peng, Jianyi Yang

Supplementary Text 1

Supplementary Tables 3

Supplementary Figures 10

Supplementary Text

Text S1: Energy function in trRosettaRNA

trRosettaRNA generates full-atom structure models by minimizing the energy defined below:

$$E = w_1 E_{dist} + w_2 E_{ori,2D} + \frac{w_2 L}{2} E_{ori,1D} + w_3 E_{cont} + w_4 E_{ros} \quad \text{(S1)}$$

where E_{dist}, $E_{ori,2D}$, $E_{ori,1D}$, E_{cont} and E_{ros} represent the 2D distance-, 2D orientation-, 1D orientation-, 2D contact-based energies and Rosetta's internal energy term, respectively; L is the length of the sequence; $w_{1\text{-}4}$ are the weights.

The 2D distances used in trRosettaRNA include five different types (Figure S1A). These distances are split into 38 bins, including 37 bins from 3 Å to 40 Å with a 1.0 Å interval and one *no-contact* bin representing the regions <3 Å and >40 Å. trRosettaRNA predicts the probability of each bin and converts the probabilities into the energy potential by the following formula:

$$score^d(b) = -ln \frac{P_b + P_B + \varepsilon}{2P_B + \varepsilon} \quad \text{(S2)}$$

where P_b is the probability for the b-th distance bin and B is the total number of bins, $\epsilon = 1E\text{-}4$ is the pseudocount parameter to avoid the singularity. Then the distance energy function can be written as:

$$E_{dist} = \sum_{d \in D} \sum_{i,j \in S_d} score_{i,j}^d \left(bin(d_{i,j})\right) \quad \text{(S3)}$$

where D is the set of defined 2D distances (Figure S1A); S_d is the set of nucleotide pairs with probability P(d<40 Å)>0.45; $d_{i,j}$ is the distance between the i-th and the j-th nucleotides; *bin*() is to convert the distance values into bins.

The 2D orientations used here include two planar angles and three dihedral angles (Figure S1C). And the 1D orientations (Figure S1B) include two planar angles and two dihedral angles. These planar/dihedral angles are binned into 12/24 segments (15° each) plus one bin referring to the P-P distance <3 Å or >40 Å. The predicted probabilities of angle bins can be converted into the energy potential by the following equation:

$$score^o(b) = -ln(P_b + \varepsilon) \quad \text{(S4)}$$

Then the 2D/1D orientation energy functions can be respectively written as:

$$E_{ori,1D} = \sum_{o \in O_1} \sum_{i=1}^{L} score^o \left(bin(o_i)\right) \quad \text{(S6)}$$

$$E_{ori,2D} = \sum_{o \in O_2} \sum_{i,j \in S_o} score^o \left(bin(o_{i,j})\right) \quad \text{(S5)}$$

where O_1 and O_2 are the sets of defined 1D and 2D orientations (Figures S1B, C); S_o is the set of nucleotide pairs with probability P(P-distance<40 Å)>0.65; $o_{i,j}$ is the 2D orientation between the i-th and j-th nucleotides; o_i is the 1D orientation corresponding to the i-th nucleotide; *bin*() is to convert the orientation values into bins.

Two nucleotides are in contact if their distance is lower than 8 Å. For the nucleotide pairs with predicted contact probabilities more than 0.6 (i.e., $P(d_{i,j}$<8) > 0.6), we define an additional energy term:

$$
cont^{d}(x)=\begin{cases}-5, & x\le d_{cut}\\ -\frac{5}{2}\left[1-sin\left(\frac{x-\left(\frac{d_{cut}+D_1}{2}\right)}{D_1-d_{cut}}\pi\right)\right], & d_{cut}<x\le D_1\\ \frac{5}{2}\left[1+sin\left(\frac{x-\left(\frac{D_2+D_1}{2}\right)}{(D_2-D_1)}\pi\right)\right], & D_1<x\le D_2\\ 5, & x>D_2\end{cases} \tag{S7}
$$

where d is one of the distance types; d_{cut} is 15 Å for the P-P distance and 20 Å for other distances; D_1 and D_2 are 35 Å and 110 Å, respectively.

Then the energy functions for 2D contacts can be written as:

$$
E_{cont}=\sum_{d\in D}\sum_{i,j\in S_c} cont^{d}\left(d_{i,j}\right) \tag{S8}
$$

where D is the set of defined 2D distances (see Figure S1A); $d_{i,j}$ is the distance between the i-th and j-th nucleotides; S_c is the set of contact nucleotide pairs with probability $P(d_{i,j}<8)$ higher than 0.6.

Supplementary Tables

Table S1. Results for 8 RNAs for which SPOT-RNA failed to predict accurate secondary structures (i.e., F1-score < 0.5). The PDB IDs in bold font are the RNAs for which the secondary structures of trRosettaRNA models are more accurate than those predicted by SPOT-RNA.

PDB ID	F1-score		RMSD (Å)			eRMSD (Å)	
	SPOT-RNA	Exacted from trRosettaRNA model	SimRNA	RNAComposer	trRosettaRNA	Model from SPOT-RNA SS	Model from native SS
**5T83**	0.37	0.43	19.5	32.3	8.7	12.1	8.4
5ZEB	0.04	0.04	19.5	14.2	5.8	8.3	10.0
**6FZ0**	0.20	0.36	25.0	21.7	13.7	13.2	11.0
**6HAG**	0.41	0.55	19.9	29.7	11.1	10.2	7.8
**6UFJ**	0.45	0.50	17.7	21.3	10.5	17.1	12.2
7A5F	0.30	0.24	14.4	10.4	10.0	6.4	7.1
**7KJU**	0.46	0.48	13.3	19.9	3.5	6.2	3.5
**7O7Y**	0.33	0.36	17.8	17.9	17.6	6.6	5.3

Table S2. Information of 20 RNA-Puzzles targets.

Date group	RNA-Puzzles ID	PDB ID	Release date	Length	N_{eff}	SS F1-score
2010-12~2013-07	PZ1	3MEI	2011-01-26	49	16	0.97
2013-07~2016-07	PZ5	4P9R	2014-05-28	188	5	0.59
	PZ10	4LCK	2013-07-31	96	620	0.76
	PZ12	4QLM	2014-08-13	125	1695	0.73
	PZ13	4XW7	2015-09-09	71	410	0.77
	PZ14Bound	5DDP	2015-12-23	61	59	0.89
	PZ14Free	5DDO	2015-12-23	61	75	0.32
	PZ15	5DI4	2015-10-07	71	186	0.59
2016-07~2019-04	PZ11	5LYS	2017-01-25	57	349	0.7
	PZ17	5K7C	2016-07-13	62	54	0.67
	PZ19	5T5A	2017-03-08	65	54	0.61
	PZ20	5Y87	2017-11-22	71	41	0.76
	PZ21	5NWQ	2017-10-18	41	20	0.67
After 2019-04	PZ22	6JQ5	2019-06-12	82	48	0.72
	PZ23	6E8U	2019-04-17	37	4	0.92
	PZ25	6P2H	2019-10-23	69	677	0.90
	PZ27	6POM	2019-11-20	170	2640	0.84
	PZ29	6TB7	2020-09-30	52	32	0.87
	PZ30	7BG9	2021-04-28	88	56	0.29
	PZ33	7ELP	2021-06-30	46	32	0.79
Average				78	354	0.72

Table S3. Results for 20 RNA-Puzzles targets. The models with RMSD < 4 Å are highlighted in bold. trRosettaRNA is denoted by trRNA.

RNA-Puzzles ID	RMSD of the first model (Å)			Best RMSD of five submitted models (Å)		
	Das	PZ_best	trRNA	Das	PZ_best	trRNA
PZ1	**4.0**	**4.0**	**3.1**	**3.4**	**3.4**	**3.1**
PZ5	10.3	10.3	20.3	9.4	9.4	18.7
PZ10	8.2	8.2	17.5	6.0	6.0	17.5
PZ11	8.5	6.0	6.7	8.3	5.0	6.6
PZ12	13.9	13.5	12.3	12.8	11.4	12.3
PZ13	7.2	7.2	10.1	5.6	5.6	9.9
PZ14Bound	12.3	5.9	9.6	9.8	5.1	9.6
PZ14Free	6.9	6.7	15.9	6.7	6.7	13.9
PZ15	-	7.1	7.8		7.1	7.8
PZ17	8.6	5.2	11.9	7.2	5.2	11.9
PZ19	15.3	5.5	8.5	9.0	5.5	8.5
PZ20	6.5	5.1	5.1	5.7	4.6	5.1
PZ21	5.7	4.1	5.7	4.1	**4.0**	5.6
PZ22	11.4	11.4	10.0	11.4	11.4	9.9
PZ23	11.2	10.8	13.4	11.2	10.6	13.1
PZ25	5.7	**2.7**	4.3	**3.5**	**2.6**	4.2
PZ27	14.4	12.8	14.8	11.6	11.0	14.8
PZ29	-	4.3	7.5	5.6	4.3	7.4
PZ30	-	5.0	19.0		5.0	14.0
PZ33	7.9	**3.8**	6.7	4.8	**3.8**	6.7
Average	-	7.0	10.5	-	6.4	10.0

Table S4. Comparisons of DI, INF, and MolProbity clash score between trRosettaRNA (denoted by trRNA) and Das on 17 RNA-Puzzles targets. ↑ means higher is better. ↓ means lower is better. DI and INF are calculated using the RNA_assessment package [1]. MolProbity clash scores are calculated using the Molprobity webserver (http://molprobity.biochem.duke.edu/).

RNA Puzzles ID	DI_ALL ↓		INF_ALL ↑		INF_WC ↑		INF_NWC ↑		INF_STACK ↑		Clash Score ↓	
	trRNA	Das	trRNA	Das	trRNA	Das	trRNA	Das	trRNA	Das	trRNA	Das
PZ1	3.6	4.3	0.86	0.92	0.90	0.95	-	-	0.85	0.91	5.7	0.0
PZ5	33.1	12.7	0.61	0.80	0.61	0.92	0.09	0.33	0.64	0.78	4.5	14.1
PZ10	27.0	9.2	0.65	0.82	0.50	0.92	0.00	0.70	0.68	0.81	1.0	19.1
PZ11	9.4	11.5	0.71	0.74	0.76	0.93	0.00	0.00	0.73	0.74	2.7	11.0
PZ12	19.1	18.4	0.64	0.75	0.69	0.90	0.24	0.40	0.67	0.71	8.7	13.7
PZ13	13.7	9.3	0.74	0.77	0.69	0.86	0.00	0.00	0.77	0.75	5.6	7.4
PZ14 Bound	12.6	15.6	0.76	0.76	0.84	0.87	0.35	0.67	0.76	0.71	2.5	7.1
PZ14 Free	20.9	7.8	0.76	0.80	0.81	0.92	0.00	0.91	0.75	0.74	2.0	16.2
PZ17	18.2	10.9	0.65	0.79	0.66	0.91	0.00	0.00	0.69	0.73	1.5	6.5
PZ19	11.9	21.6	0.71	0.71	0.69	0.85	0.00	0.33	0.73	0.65	3.8	15.0
PZ20	6.3	8.2	0.81	0.80	0.88	0.89	0.22	0.35	0.80	0.77	3.1	16.9
PZ21	8.8	8.9	0.65	0.64	0.70	0.84	0.13	0.00	0.67	0.70	2.3	18.8
PZ22	15.2	17.8	0.65	0.64	0.67	0.69	0.00	0.00	0.69	0.67	4.2	3.8
PZ23	28.1	20.0	0.48	0.56	0.87	0.75	0.00	0.34	0.56	0.61	1.7	6.7
PZ25	5.9	7.4	0.72	0.78	0.73	0.95	0.11	0.45	0.77	0.74	4.5	11.3
PZ27	23.9	18.4	0.62	0.78	0.74	0.89	0.00	0.64	0.60	0.73	1.6	8.2
PZ33	9.0	11.3	0.75	0.70	0.60	0.75	0.22	0.00	0.76	0.70	2.1	8.1
Average	15.7	12.6	0.69	0.75	0.73	0.87	0.09	0.31	0.71	0.73	3.2	10.8

Table S5. Results for 12 RNA targets in CASP15. For all compared groups, we evaluate the first models for each target.

Target type	CASP ID	SPOT-RNA SS F1-score	eRMSD (Å)	RMSD of the first model (Å)					
			Yang-Server	Yang-Server	AIchemy-RNA2	Chen	RNApolis	Deep learning best*	Overall best
Natural	R1107	0.51	12.4 (3.2†)	17.9 (4.3†)	4.5	6.7	14.1	5.9	4.5
	R1108	0.70	10.3 (3.1†)	9.1 (4.8†)	5.3	6.2	13.9	5.4	5.3
	R1116	0.70	11.4	12.2	23.3	19.2	12.7	12.2	5.5
	R1117	0.54	2.4	2.7	2.3	2.5	2.7	2.7	2.3
	R1149	0.69	11.5 (9.7†)	15.2 (10.6†)	18.6	14.2	19.4	8.7	7.4
	R1156	0.56	12.5	17.7	25.3	11.0	23.7	12.9	7.5
	R1189	0.55	21.3	22.4	22.0	21.2	20.3	23.0	20.3
	R1190	0.73	20.9	22.6	23.9	18.8	23.8	23.3	18.8
	Average	0.62	11.7 (9.1†)	14.8 (11.9†)	15.7	12.5	16.3	11.8	8.9
Synthetic	R1126	0.70	23.0	38.1	8.9	52.8	20.0	30.2	8.9
	R1128	0.92	13.6	22.3	4.3	6.7	15.8	22.1	4.3
	R1136	0.73	27.4	46.2	8.2	14.3	12.7	33.4	8.2
	R1138	0.79	43.5	49.5	21.8	12.3	11.8	35.5	11.8
	Average	0.79	26.9	39.0	10.8	21.5	15.1	30.3	8.3
Overall average		0.70	17.2 (15.5†)	22.9 (20.9†)	14.0	15.5	15.9	17.9	8.7

*According to the CASP15 abstracts (https://predictioncenter.org/casp15/doc/CASP15_Abstracts.pdf), there are 14 RNA prediction groups utilizing deep learning-based methods to predict RNA structures: AIchemy_RNA, BAKER, CoMMiT-human, CoMMiT-server, DF_RNA, GWxraylab, Graphen_Medical, Schug_Lab, UltraFold, UltraFold_Server, Yang, Yang-Multimer, Yang-Server, and rDP.

†trRosettaRNA results with secondary structure templates as inputs.

Table S6. Comparison of INF, lDDT and MolProbity clash score between Yang-Server and AIchemy_RNA2 for 12 RNA targets in CASP15. ↑ means higher is better. ↓ means lower is better. The data for Yang-Server and AIchemy_RNA2 are collected from the CASP15 official repository (https://github.com/DasLab/casp-rna) [2]. MolProbity clash scores for refined Yang-Server models are calculated using the Molprobity webserver (http://molprobity.biochem.duke.edu/).

Target ID	INF_ALL ↑		lDDT ↑		Clash Score ↓		
	Yang-Server	AIchemy_RNA2	Yang-Server	AIchemy_RNA2	Yang-Server	Refined Yang-Server	AIchemy_RNA2
R1107	0.59	0.87	0.41	0.72	35.73	2.71	14.93
R1108	0.76	0.88	0.56	0.75	34.36	3.62	15.37
R1116	0.77	0.87	0.66	0.68	53.24	4.17	10.13
R1117	0.70	0.80	0.61	0.75	61.65	6.27	35.53
R1126	0.74	0.86	0.50	0.69	21.99	1.55	14.52
R1128	0.86	0.93	0.72	0.87	61.11	1.32	13.29
R1136	0.75	0.94	0.56	0.78	40.96	3.66	13.98
R1138	0.74	0.92	0.56	0.74	49.87	3.47	16.12
R1149	0.82	0.88	0.64	0.71	7.06	2.52	20.93
R1156	0.75	0.88	0.59	0.70	36.49	2.31	14.78
R1189	0.70	0.67	0.54	0.50	1.57	2.61	18.28
R1190	0.72	0.67	0.58	0.55	2.09	2.35	12.27
Average	0.74	0.85	0.58	0.70	33.84	3.05	16.68

Table S7. Impact of the different restraints on the structure modeling accuracy on 20 RNA-Puzzles targets. Source data are provided as a Source Data file.

Energy terms	RMSD (Å)
2D distances	11.34
2D distances + 2D orientations	11.13
2D distances + 2D orientations + 1D orientations	10.79
2D distances + 2D orientations + 1D orientations + 2D contacts	10.51

Supplementary Figures

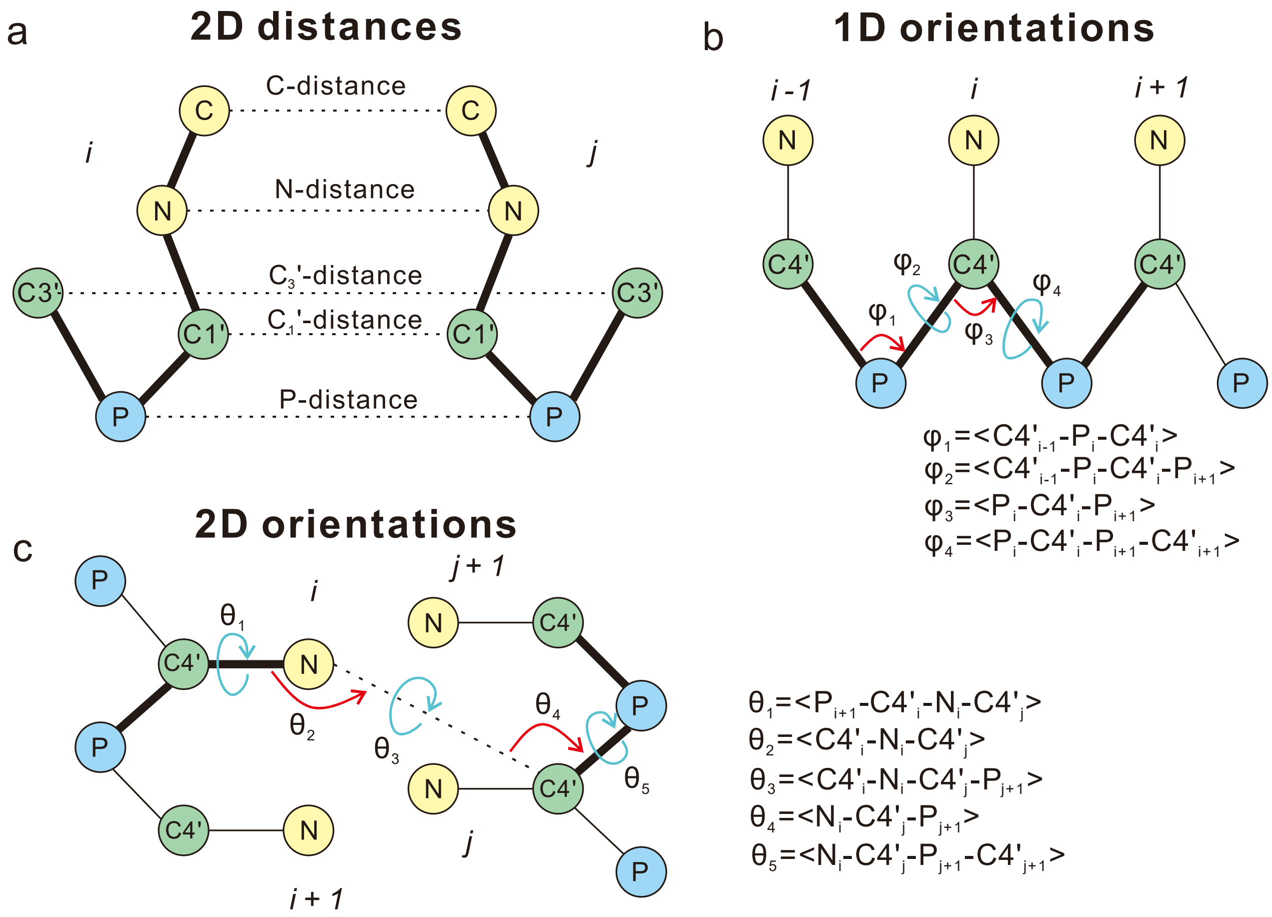

Figure S1. Definition of the 1D and 2D geometries in trRosettaRNA. (a) 2D distances. (b) 1D orientations. (c) 2D orientations. N refers to the N9 atom for purine and the N1 atom for pyrimidine. C refers to C2 atom for purine and C4 atom for pyrimidine. *i*, *j* are the indices of nucleotides.

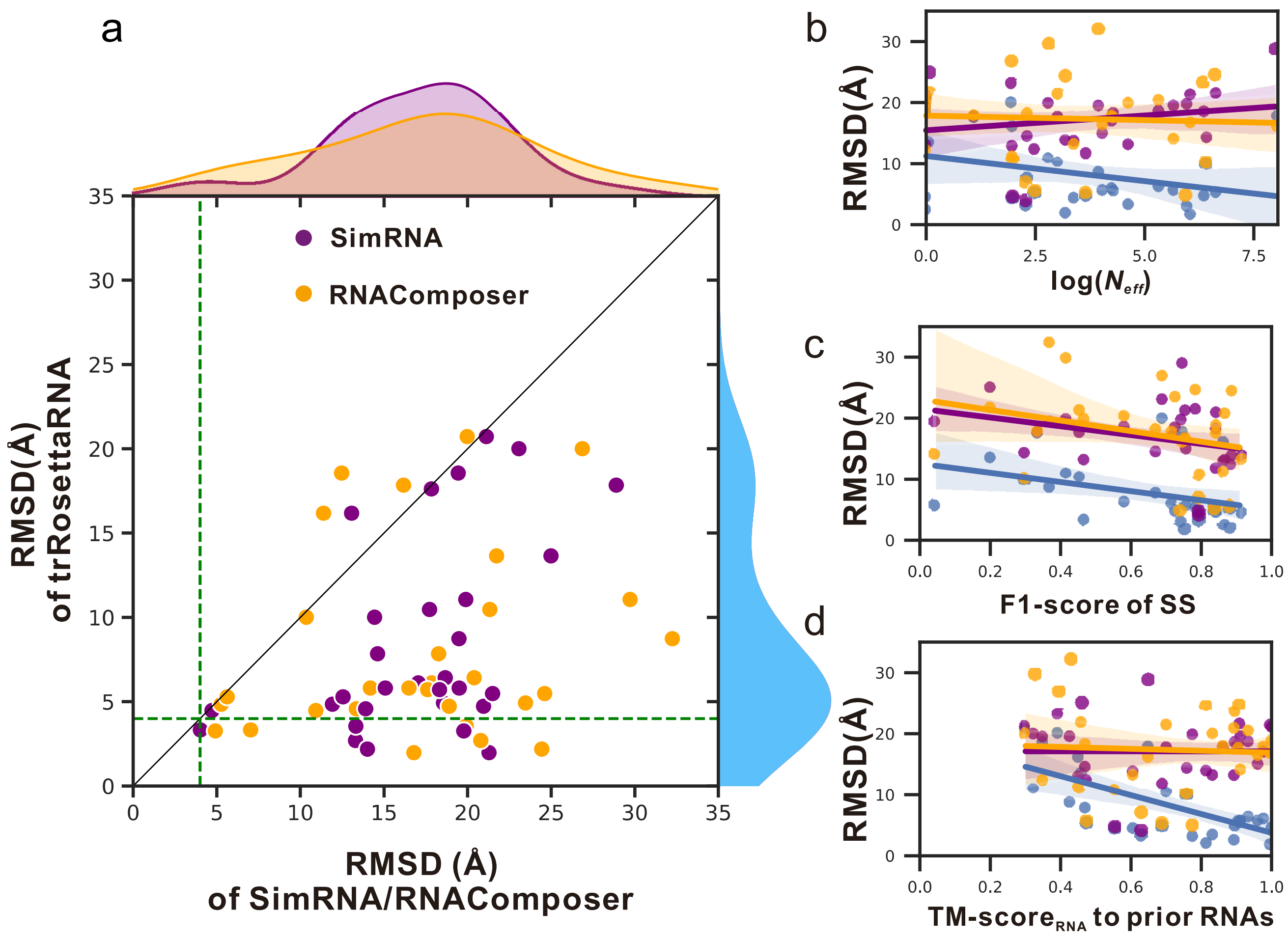

Figure S2. Performance on 30 independent RNAs. (a) head-to-head comparison between trRosettaRNA and two representative methods, SimRNA and RNAComposer (n=30 RNAs). The dashed horizontal and vertical lines correspond to an RMSD of 4 Å. The bar plots show the RMSD distributions. (b) the RMSD as a function of the logarithm of the MSA depth (N_{eff}). (c) RMSD as a function of the F1-score of the predicted secondary structure (denoted by SS). (d) RMSD as a function of the maximum TM-score$_{RNA}$ to prior RNAs. The gray and black dash lines in (d) refer to the TM-score$_{RNA}$ thresholds of 0.45 and 0.6 (homology match and very good homology match) respectively. The blue, purple, and orange dots in B-D refer to trRosettaRNA, SimRNA, and RNAComposer, respectively. Source data are provided as a Source Data file.

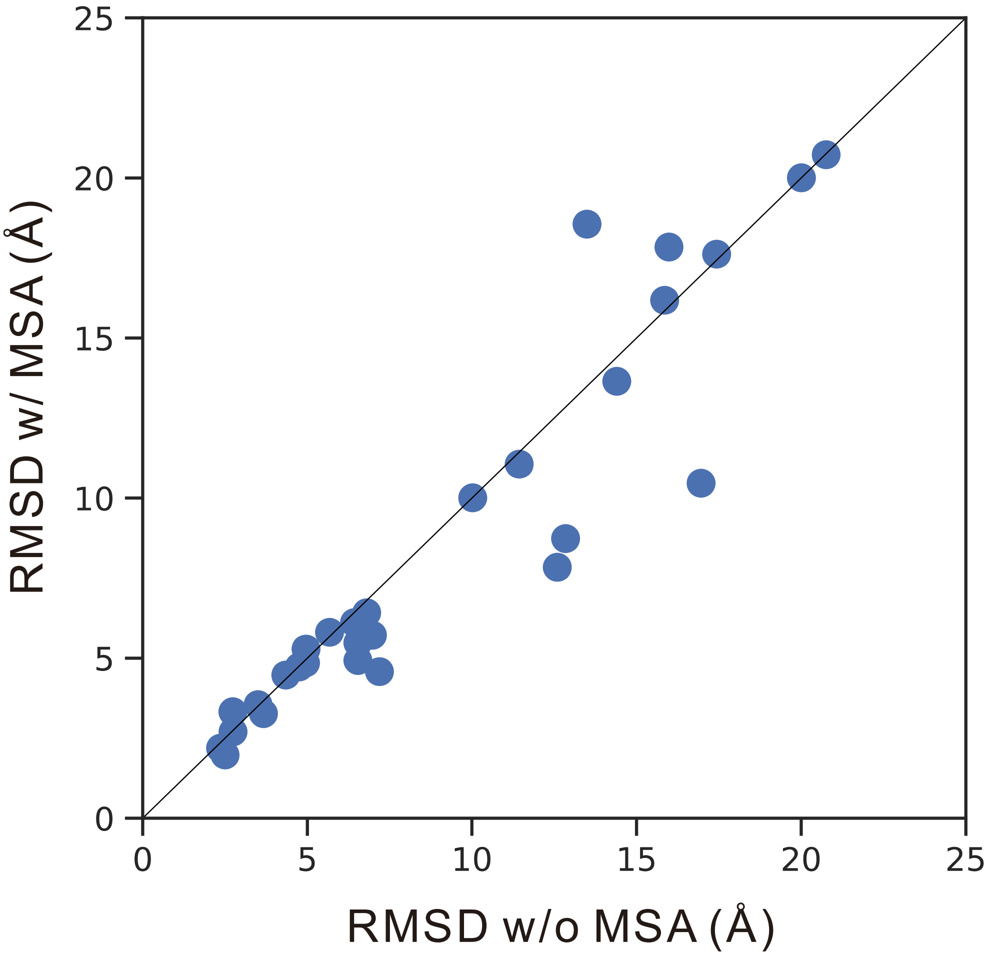

Figure S3. Analysis of MSA's contribution to RNA structure prediction. Head-to-head comparison between the RMSDs of trRosettaRNA models predicted with and without MSA (n=30 independent RNAs). Source data are provided as a Source Data file.

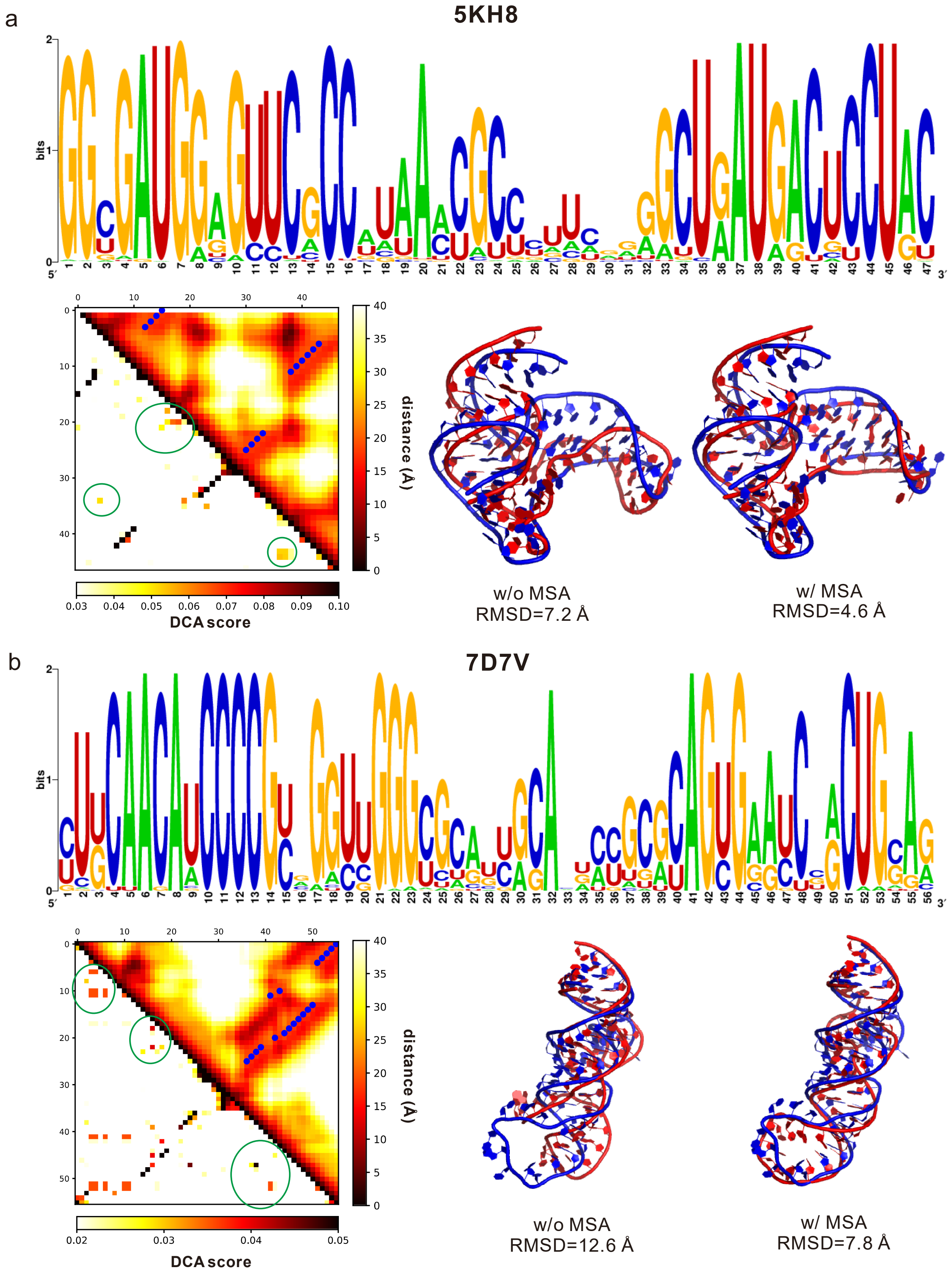

Figure S4. Two examples to illustrate the contribution of MSA. The two examples are (a) 5KH8 and (b) 7D7V. For each example, the following items are presented: MSA sequence log plotted by WebLogo [3], the direct couplings analysis (DCA) matrix of the MSA calculated using PLMC [4] (located in the lower left of the 2D map), the experimental distance map (located in the upper right of the 2D map) and the superposition of the predicted structures (red) with the experimental structures (blue).

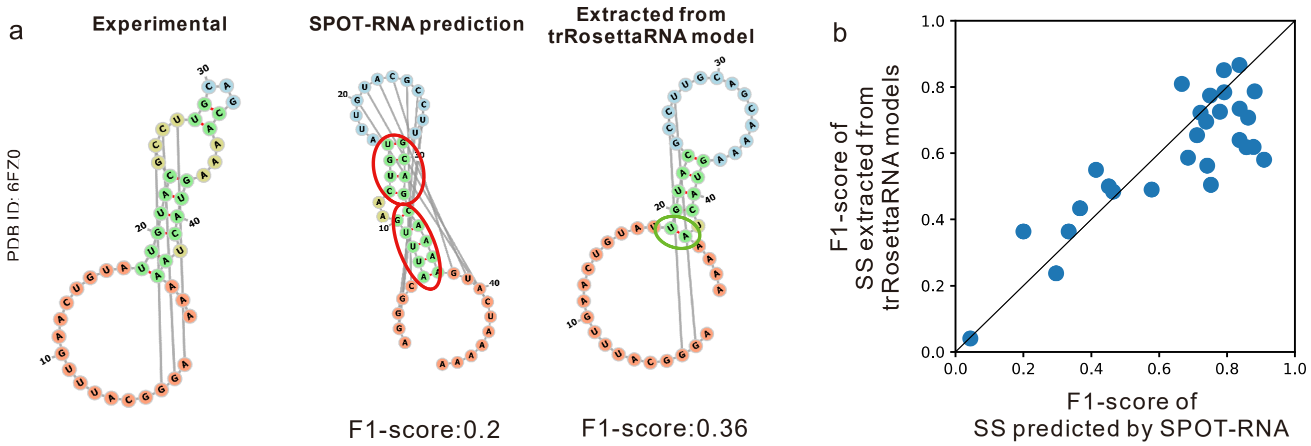

Figure S5. Comparison between the derived/predicted secondary structures. (a) the experimental and predicted secondary structures of an example RNAs on which the SPOT-RNA predictions are inaccurate (i.e., F1-score < 0.5). (b) the head-to-head comparison between the secondary structures (denoted by SS) extracted from trRosettaRNA models and those predicted by SPOT-RNA in terms of F1-score (n=30 independent RNAs). Source data are provided as a Source Data file.

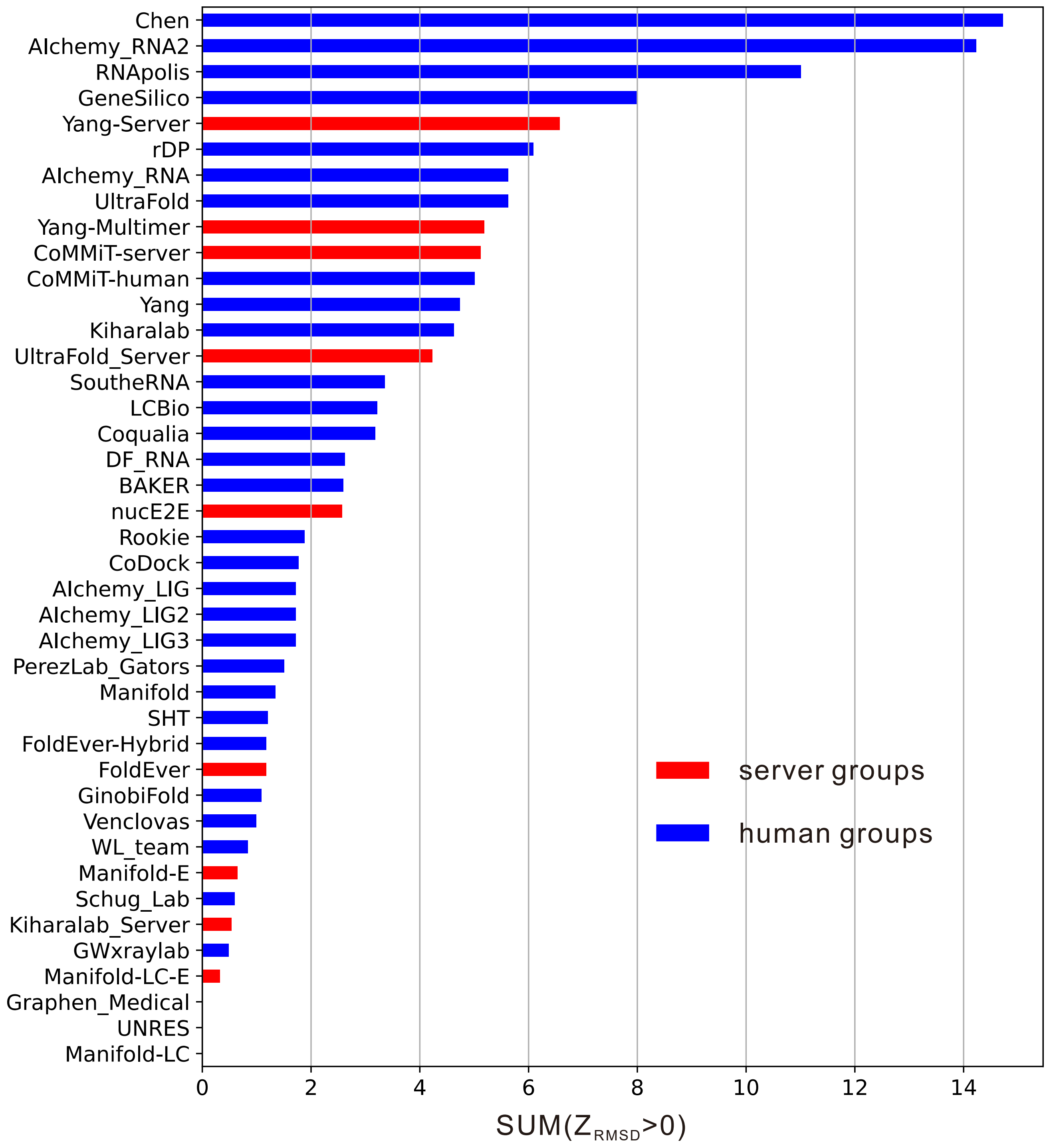

Figure S6. The performance ranking of CASP15 RNA structure prediction groups based on the cumulative Z-score of RMSD. The cumulative Z-score is calculated following the CASP official procedure: 1) calculate Z-scores based on the negative RMSD for all first-submitted models; 2) remove the models with Z-scores below the tolerance threshold (set to -2.0); 3) recalculate Z-scores on the reduced dataset; 4) assign Z-scores below the penalty threshold (set to 0.0) to the value of this threshold. Source data are provided as a Source Data file. Note that this RMSD-based ranking is calculated on our own but is largely consistent with the assessor's version (please see p10 in Dr. Rhiju Das' slides: https://predictioncenter.org/casp15/doc/presentations/Day3/Assessment_RNA-CASP_RDas.pdf).

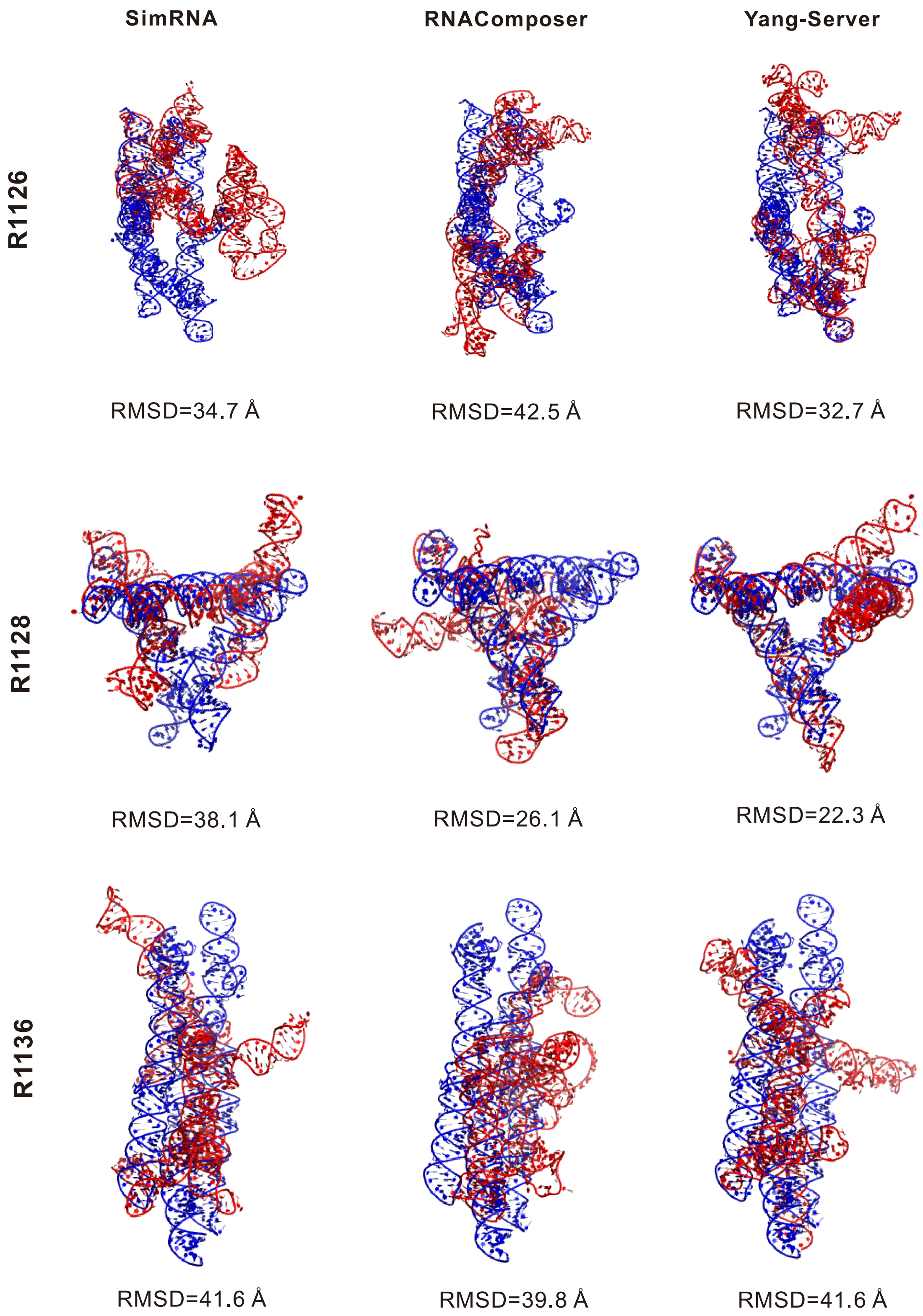

Figure S7. Comparison of 3D modelling results for synthetic RNAs in CASP15 between Yang-Server and representative automated methods. Both predicted 3D structures (in the red cartoon) are superimposed onto the experimental structures (in the blue cartoon).

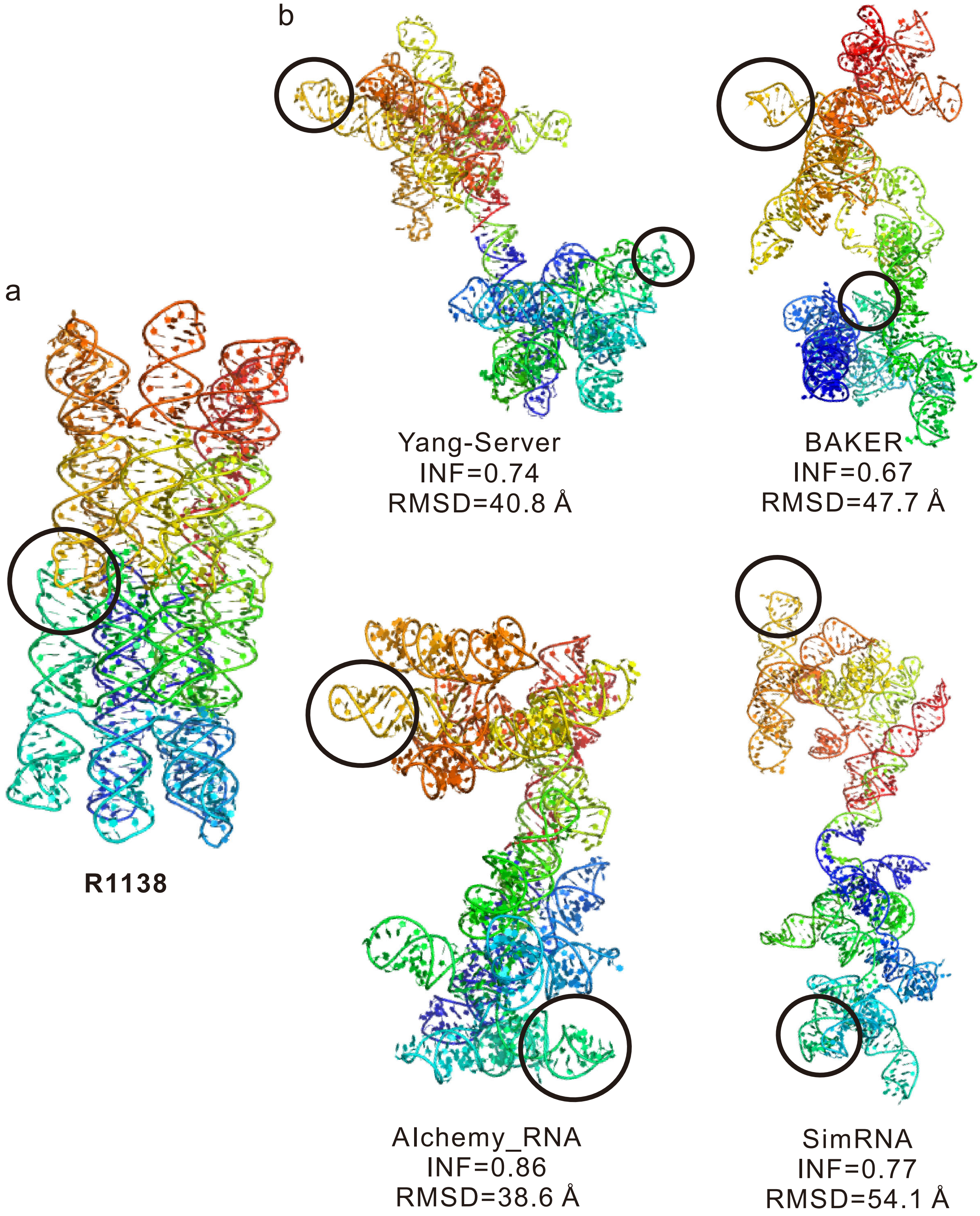

Figure S8. Results for an example synthetic RNA (R1138) from CASP15 to illustrate the challenge for automated modeling of synthetic RNAs in CASP15. (a) the experimental structure of R1138 (mature state). (b) the structures predicted by representative automated methods. The SimRNA model was generated by running its standalone package locally, utilizing the same secondary structure as the one used by Yang-Server. The models from other methods represent the best submissions made by their respective groups during the CASP15 season.

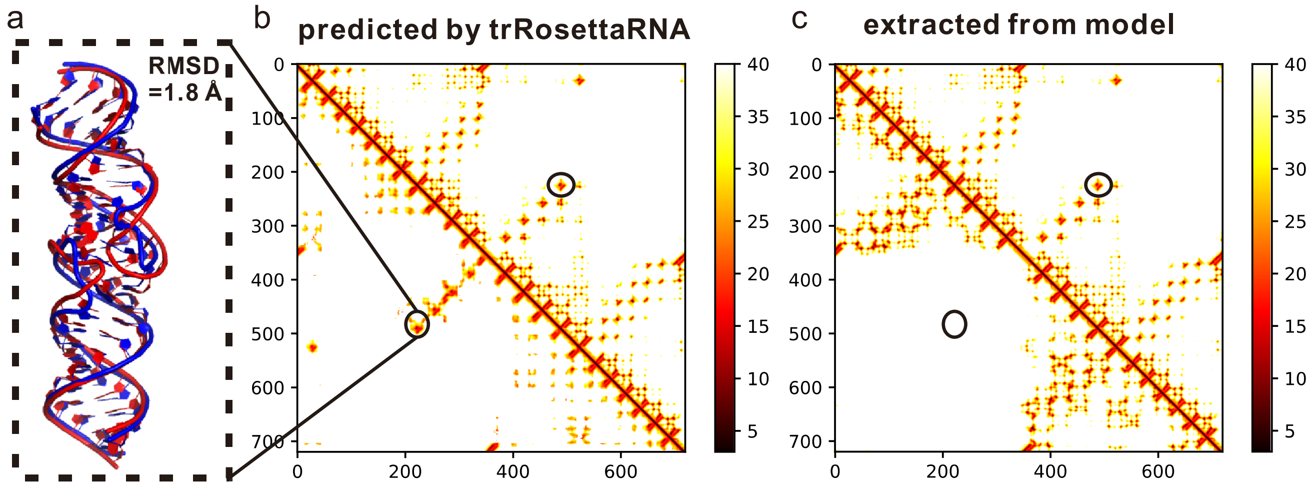

Figure S9. Analysis of the highlighted kissing loop in R1138. (a) the trRosettaRNA result by exclusively modeling the highlighted kissing loop between residues 214~240 and residues 477~505. During the modeling process, a connecting linker composed of 50 Adenines was introduced, which was subsequently removed upon completion of the modeling procedure. The trRosettaRNA model (red cartoon) is shown and superposed to the corresponding motif (blue cartoon) extracted from the experimental structure of R1138. (b) the comparison between distance maps predicted by trRosettaRNA (lower left) and extracted from the experimental structure (upper right). (c) the comparison between distance maps extracted from the trRosettaRNA model (lower left) and the experimental structure (upper right). The black circles in (b) and (c) correspond to the kissing loop highlighted in Figure S8.

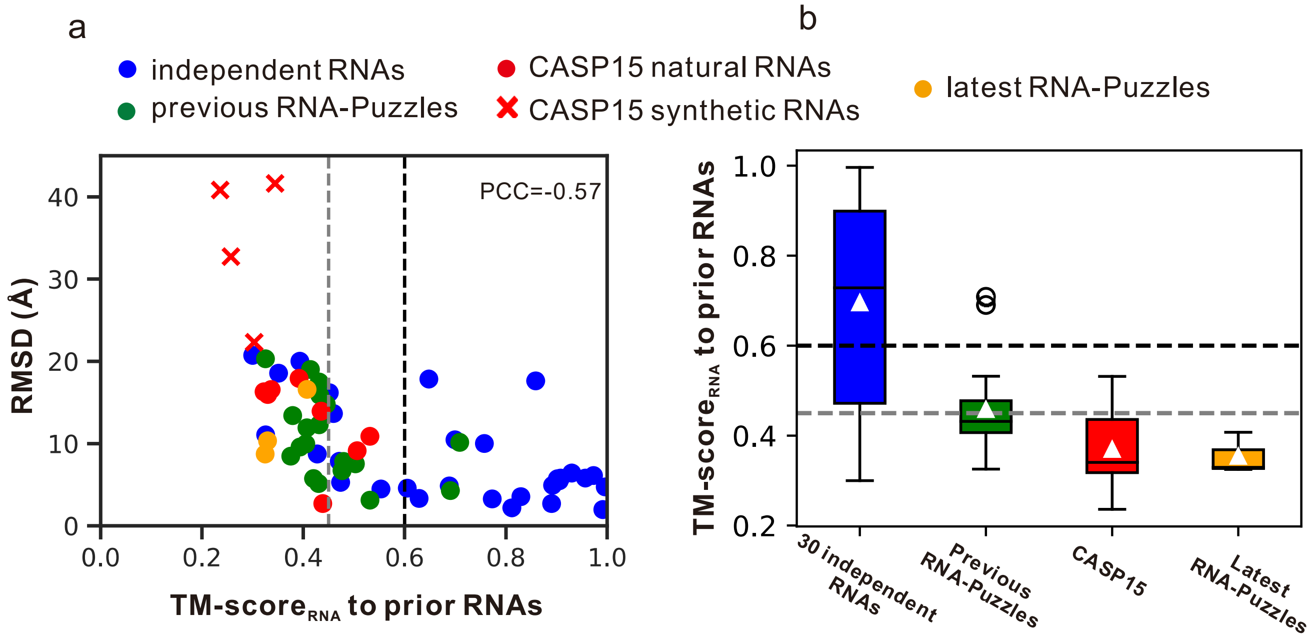

Figure S10. Analysis of modeling difficulty for RNAs from benchmarks and blind tests. (a) relationship between RMSD and the maximum TM-score$_{RNA}$ to prior RNAs (n=65 RNAs). (b) boxplot illustrating the distributions of the maximum TM-score$_{RNA}$ to prior RNAs on different datasets. The central line in each box represents the median, while the box spans the interquartile range (IQR; the range between the 75th percentile and the 25th percentile of the data). Whiskers extend to data points within the whisker length (i.e., 1.5 times the IQR), and outliers are shown as individual points outside this range. Mean values are indicated by white triangles. Source data are provided as a Source Data file.

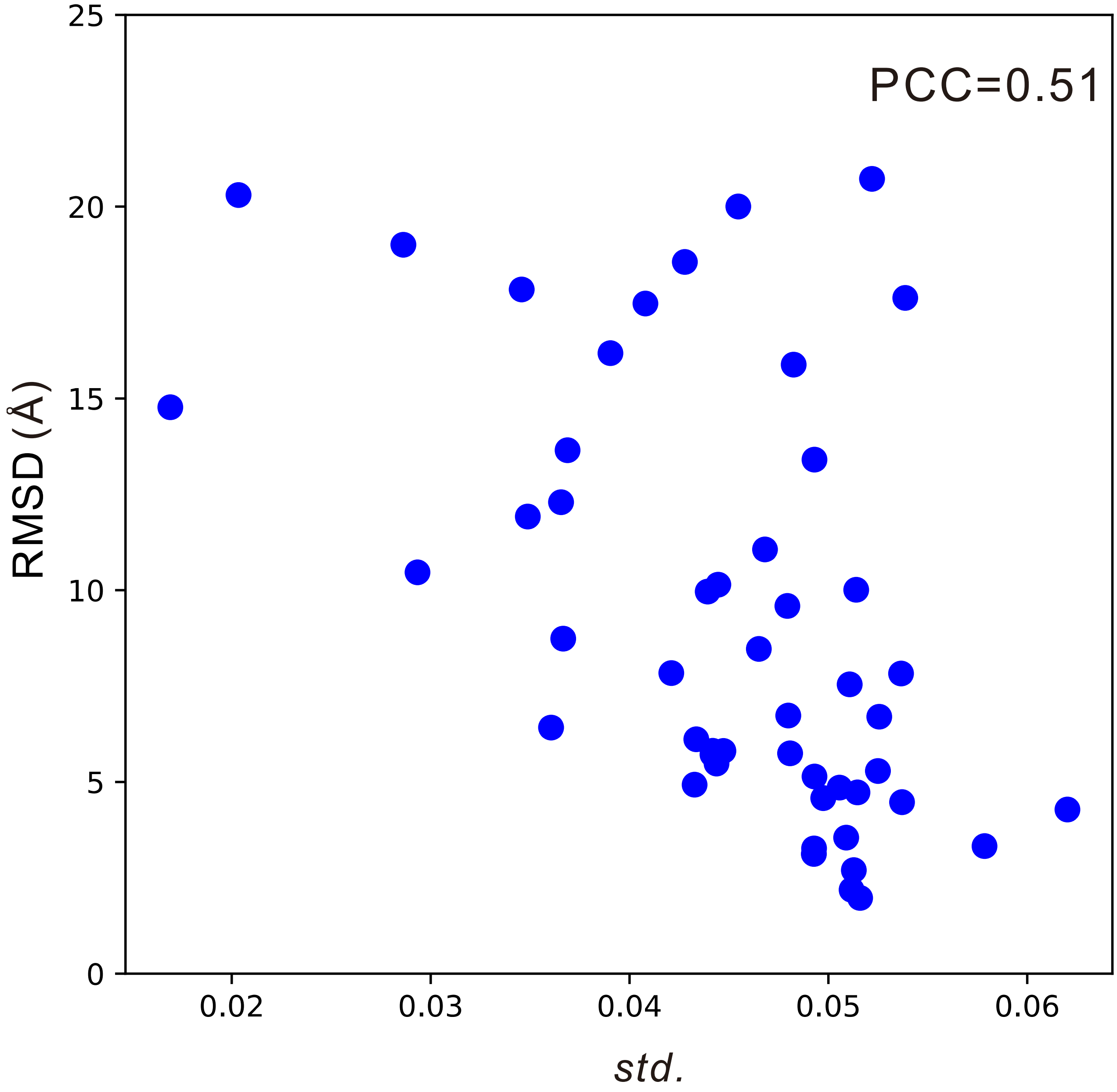

Figure S11. Relationship between RMSD and the average standard deviations of the predicted distance distributions (n=50 RNAs). Source data are provided as a Source Data file.

Supplementary References

1. Magnus, M. *et al.* RNA-Puzzles toolkit: a computational resource of RNA 3D structure benchmark datasets, structure manipulation, and evaluation tools. *Nucleic Acids Research* **48**, 576-588 (2020).
2. Rhiju, D. *et al.* Assessment of three-dimensional RNA structure prediction in CASP15. *bioRxiv*, 2023.2004.2025.538330 (2023).
3. Crooks, G.E., Hon, G., Chandonia, J.-M. & Brenner, S.E. WebLogo: A Sequence Logo Generator. *Genome Research* **14**, 1188-1190 (2004).
4. Weinreb, C. *et al.* 3D RNA and Functional Interactions from Evolutionary Couplings. *Cell* **165**, 963-975 (2016).
