# Supplementary material for: trRosettaRNA: automated prediction of RNA 3D structure with transformer network
Source: Nat Commun. 2023 Nov 9;14:7266. doi: 10.1038/s41467-023-42528-4 (PMC10636060; doi:10.1038/s41467-023-42528-4)
Supplement: Supplementary file 3 — Reporting Summary [file 41467_2023_42528_MOESM3_ESM.pdf]

nature portfolio

Corresponding author(s): Jianyi Yang, Zhenling Peng, Fa Zhang

Last updated by author(s): Sep 29, 2023

Reporting Summary

Nature Portfolio wishes to improve the reproducibility of the work that we publish. This form provides structure for consistency and transparency in reporting. For further information on Nature Portfolio policies, see our Editorial Policies and the Editorial Policy Checklist.

Statistics

For all statistical analyses, confirm that the following items are present in the figure legend, table legend, main text, or Methods section.

n/a	Confirmed	
☐	☒	The exact sample size (*n*) for each experimental group/condition, given as a discrete number and unit of measurement
☐	☒	A statement on whether measurements were taken from distinct samples or whether the same sample was measured repeatedly
☐	☒	The statistical test(s) used AND whether they are one- or two-sided<br>*Only common tests should be described solely by name; describe more complex techniques in the Methods section.*
☒	☐	A description of all covariates tested
☒	☐	A description of any assumptions or corrections, such as tests of normality and adjustment for multiple comparisons
☐	☒	A full description of the statistical parameters including central tendency (e.g. means) or other basic estimates (e.g. regression coefficient) AND variation (e.g. standard deviation) or associated estimates of uncertainty (e.g. confidence intervals)
☐	☒	For null hypothesis testing, the test statistic (e.g. *F*, *t*, *r*) with confidence intervals, effect sizes, degrees of freedom and *P* value noted<br>*Give P values as exact values whenever suitable.*
☒	☐	For Bayesian analysis, information on the choice of priors and Markov chain Monte Carlo settings
☒	☐	For hierarchical and complex designs, identification of the appropriate level for tests and full reporting of outcomes
☐	☒	Estimates of effect sizes (e.g. Cohen's *d*, Pearson's *r*), indicating how they were calculated

Our web collection on statistics for biologists contains articles on many of the points above.

Software and code

Policy information about availability of computer code

Data collection	CD-HIT (version 4.8.1) was used to remove redundant sequences in test set and distillation set. BLAST+ (version 2.7.1) was used to remove the tested RNAs redundant to the training sets. SPOT-RNA (version 1.0) was used to predict secondary structures. Infernal (version 1.1.4) and rMSA (version 1.0) were used to generate MSA. R2DT (version 1.0) was used to detect secondary structures templates. The source code of trRosettaRNA are available at Zenodo (doi: 10.5281/zenodo.8388687) and our website (https://yanglab.qd.sdu.edu.cn/trRosettaRNA/).
Data analysis	PyRosetta4 (version 2019.23) was used to perform energy minimization. RNA-Puzzles toolkit (version 1.0) and RNAalign (version 1.0) were used to evaluate the predicted structures. PyMOL (version 2.5.2) was used to display the 3D structures. WebLogo (version 2.8.2) was used to generate sequence log for MSA. PLMC (version 1.0) was used to calculate coevolutionary information for MSA. forna (version 1.0) was used to visualize RNA secondary structures.

For manuscripts utilizing custom algorithms or software that are central to the research but not yet described in published literature, software must be made available to editors and reviewers. We strongly encourage code deposition in a community repository (e.g. GitHub). See the Nature Portfolio guidelines for submitting code & software for further information.

Data

Policy information about availability of data

All manuscripts must include a data availability statement. This statement should provide the following information, where applicable:

- Accession codes, unique identifiers, or web links for publicly available datasets
- A description of any restrictions on data availability
- For clinical datasets or third party data, please ensure that the statement adheres to our policy

The training sets, the set of 20 RNA-Puzzles RNAs, and the set of 30 independent RNAs can be downloaded from Zenodo 51 and our website (https://yanglab.qd.sdu.edu.cn/trRosettaRNA/). The RNAs from blind tests of CASP15 and RNA-Puzzles can be downloaded from https://predictioncenter.org/casp15/results.cgi?tr_type=rna and https://www.rnapuzzles.org/results/, respectively. The PDB entries mentioned in this study (3IVK, 5KH8, 5LYS, 6D89, 7D7V, 8DP3, 8GXC, and 8HB8) were obtained by four-digit accession codes in the Protein Data Bank repository (https://www.rcsb.org/). The sequence databases of NCBI's nt, Rfam, and RNAcentral used to generate MSA in this study can be downloaded https://www.ncbi.nlm.nih.gov/nucleotide, https://rfam.org/, and https://rnacentral.org/, respectively. The source data underlying Tables 1, S7 and Figures 4-7, S2, S3, S5, S6, S10, S11 are provided in the Source Data file. Source data are provided with this paper.

Research involving human participants, their data, or biological material

Policy information about studies with human participants or human data. See also policy information about sex, gender (identity/presentation), and sexual orientation and race, ethnicity and racism.

Reporting on sex and gender	N/A
Reporting on race, ethnicity, or other socially relevant groupings	N/A
Population characteristics	N/A
Recruitment	N/A
Ethics oversight	N/A

Note that full information on the approval of the study protocol must also be provided in the manuscript.

Field-specific reporting

Please select the one below that is the best fit for your research. If you are not sure, read the appropriate sections before making your selection.

☒ Life sciences ☐ Behavioural & social sciences ☐ Ecological, evolutionary & environmental sciences

For a reference copy of the document with all sections, see nature.com/documents/nr-reporting-summary-flat.pdf

Life sciences study design

All studies must disclose on these points even when the disclosure is negative.

Sample size	No statistical methods were used to predetermine sample sizes. The RNA-Puzzles targets were collected from RNA-Puzzles community. The independent RNAs were collected from the RNA entries released after 2017-01 in PDB database. Both test sets were filtered to remove any redundancy to the training set, resulting in 20 RNA-Puzzles targets and 30 independent RNAs. We did not choose any specific sample sizes but rather aimed to collect a sufficient number of samples to serve as fair benchmark tests.
Data exclusions	No data were excluded from the analyses.
Replication	We have run the programs 150 (=(20+30)*3) times with each protein running three replications. All replications were successful. Please follow our instructions on the web server page, or instructions in the standalone package.
Randomization	The benchmark RNAs were grouped into 20 RNA-Puzzles targets and 30 independent RNAs according to their sources (whether from the RNA-Puzzles competition).
Blinding	There was no blinding group or analysis in this manuscript as all RNAs in our test have the deposited models in the PDB.

Reporting for specific materials, systems and methods

We require information from authors about some types of materials, experimental systems and methods used in many studies. Here, indicate whether each material, system or method listed is relevant to your study. If you are not sure if a list item applies to your research, read the appropriate section before selecting a response.

Materials & experimental systems

n/a	Involved in the study
☒	☐ Antibodies
☒	☐ Eukaryotic cell lines
☒	☐ Palaeontology and archaeology
☒	☐ Animals and other organisms
☒	☐ Clinical data
☒	☐ Dual use research of concern
☒	☐ Plants

Methods

n/a	Involved in the study
☒	☐ ChIP-seq
☒	☐ Flow cytometry
☒	☐ MRI-based neuroimaging
